# Role of Deep Learning in Plastic Surgery: Transforming Art With Intelligence

**DOI:** 10.7759/cureus.99329

**Published:** 2025-12-15

**Authors:** Mohd Altaf Mir

**Affiliations:** 1 Burns & Plastic Surgery, All India Institute of Medical Sciences, Bathinda, Bathinda, IND

**Keywords:** aesthetic surgery, artificial intelligence, deep learning, machine learning, plastic surgery, reconstruction

## Abstract

Deep learning, a branch of artificial intelligence (AI), is revolutionizing modern medicine through its capacity for pattern recognition, data-driven decision support, and predictive analytics. In plastic surgery, a specialty that merges precision with artistry, deep learning is emerging as a transformative tool. From reconstructive planning and aesthetic simulations to postoperative monitoring and skill assessment, AI is reshaping the scope of surgical innovation. However, challenges related to data quality, ethics, and integration into clinical workflows remain. This editorial explores the expanding role of deep learning in plastic surgery and emphasizes its potential to augment, rather than replace, surgical expertise.

## Editorial

Plastic surgery stands at the threshold of a technological renaissance, catalyzed by the proliferation of artificial intelligence (AI), most notably, deep learning [1]. Our specialty, long rooted in anatomical mastery and aesthetic discernment, is now enriched by the arrival of machine-learned patterns, multilayered neural networks, and data-fed precision [2,3]. The result is a field poised for transformation, where surgical artistry finds its newest ally in computational intelligence.

In the past decade, the impact of deep learning in plastic surgery has been evidenced across the entire clinical continuum. Consider reconstructive surgery, where convolutional neural networks accurately classify burn depth and predict flap viability, often performing at or above the level of expert clinicians [4-6]. Major multicenter trials now routinely demonstrate diagnostic accuracy rates above 85% for floridly complex tasks such as thermal imaging analysis and early detection of flap compromise, the foundation for earlier intervention and reduced morbidity [5-7]. Even the once subjective evaluation of tissue viability is increasingly being standardized by unbiased algorithms, recordable in real-time and accessible even in resource-limited settings.

A similarly remarkable transformation is seen in the aesthetic and craniofacial disciplines. AI-powered facial analysis tools objectify symmetry, help quantify facial proportion, and support preoperative simulation, enhancing communication and expectation management with our patients [6-8]. These technologies break the traditional boundaries of subjective assessment, empowering both surgeons and patients through quantifiable, reproducible measurements. Generative adversarial networks can now simulate postoperative outcomes, giving patients a tangible vision of surgical results, an innovation shown to improve satisfaction and shared decision-making [7-9].

Education, a linchpin of surgical progress, is likewise evolving. Deep learning-driven motion analysis translates hands-on performance into objective feedback, scaffolding modern competency-based training [9,10]. AI-assisted robotic platforms, already helping to steady the surgeon's hand and expand dexterity, gesture toward a future where technology augments the reach of human capability [8-11].

The influence of deep learning extends powerfully into postoperative care as well. AI-enabled telemonitoring can now detect complications from routine smartphone images, promising earlier intervention and democratized postoperative surveillance [10,11]. For a specialty founded on nuanced, sometimes minute-to-minute clinical changes, such tools are invaluable in reducing unnecessary visits and ensuring high-quality remote care.

Yet, as with any technological leap, challenges abound. Despite successful validation in retrospective and cross-sectional studies, prospective clinical trials remain scarce, and many algorithms have yet to be externally validated across different populations [4-6]. There is a risk, too, that non-representative datasets will perpetuate bias, compromising accuracy for minority or marginalized groups [11]. The “black box” nature of many models further complicates clinical trust and ethical integration, demanding frameworks that prioritize explainability, transparency, and shared agency between surgeon, patient, and AI. Perhaps most importantly, as AI codifies aesthetic norms, our community must remain vigilant-guarding against a sterile homogeneity that could stifle individuality and cross-cultural beauty.

Nevertheless, the narrative that technology will supplant surgical artistry is fundamentally flawed. AI’s greatest value lies in its ability to amplify what makes plastic surgery exceptional: creative problem-solving, empathic patient care, and the pursuit of individualized excellence [2]. As deep learning platforms increasingly inform diagnosis, planning, and evaluation, the surgeon’s unique insights shaped by experience, conversation, and compassion will remain central to every decision.

Moving forward, responsible adoption of deep learning in plastic surgery requires rigorous validation, diverse and representative datasets, multidisciplinary collaboration, and ongoing ethical debate. Regulatory frameworks and specialty guidelines must keep pace, ensuring that innovation never outruns patient safety or equitable access. The future belongs to collaborative intelligence: teams where surgeons, scientists, engineers, and algorithms work in concert to deliver more precise, personalized, and humane care.

This is the moment to lead, not follow. As we shape the algorithmic future of plastic surgery, let us do so with humility, vigilance, and an unyielding commitment to our patients’ well-being, a commitment now emboldened by tools of unprecedented power.
